# Preparation of core/shell CdTe@hMSN for enhanced tumor vasculature-specific drug delivery

**DOI:** 10.1039/c8ra07193d

**Published:** 2018-11-20

**Authors:** Dongzhi Yang, Na Wang, Haixia Ji, Shian Sun, Jingjing Dong, Yuanyuan Zhong, Chuntong Qian, Huanghuang Xu

**Affiliations:** Jiangsu Key Laboratory of New Drug Research and Clinical Pharmacy, Xuzhou Medical University Xuzhou Jiangsu 221004 China dongzhiy@xzhmu.edu.cn +86-516-83262138; Department of Pharmaceutical Analysis, School of Pharmacy, Xuzhou Medical University Xuzhou Jiangsu 221004 China; Xuzhou Air Force College Xuzhou Jiangsu 221000 China

## Abstract

Due to excellent optical properties, CdTe quantum dots (QDs) exhibit great potential in cancer imaging. However, CdTe QDs can be quickly cleared out before reaching the desired location because of their ultra-small size. The structure and optical properties of CdTe QDs are also easily affected by the surrounding solution, which leads to their compromised applications *in vivo*. Here, CdTe QDs were incorporated into hollow mesoporous silica nanoparticles (hMSN) to form CdTe@hMSN nano-platforms. The as-synthesized system maintained the excellent emission properties of CdTe QDs; meanwhile, relatively high drug loading efficiency was also observed for doxorubicin (DOX). With the target for vascular endothelial growth factor (VEGF), the formed CdTe@hMSN(DOX)–VEGF Abs showed feasibility of tumor-oriented drug delivery and CdTe@hMSN conjugate accumulation. The high accumulation and enhanced targeted drug delivery of CdTe@hMSN conjugates in tumor nodules confirmed that CdTe@hMSN conjugates can serve as promising candidates for cancer detection and treatment.

## Abstract 2

Due to excellent optical properties and high cargo holding capacity, CdTe@hMSN exhibit great potential in cancer imaging and drug delivery.

## Graphical abstract

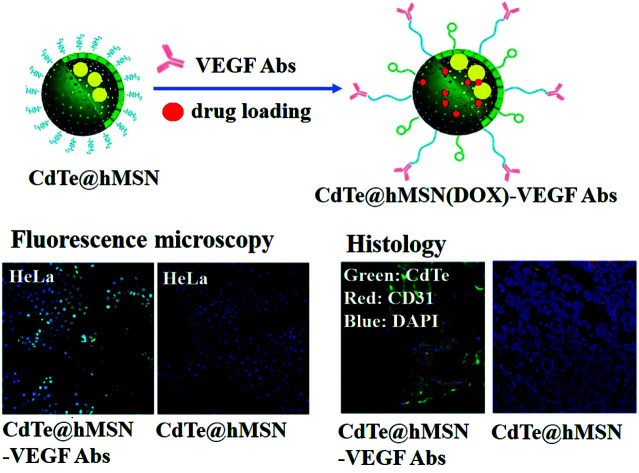

## Introduction

With the fast development of nanoscience and nanotechnology, a variety of nanoparticles have been developed as drug delivery systems^1–3^ due to their unique properties such as tunable size, well-defined optical and surface properties and excellent biocompatibility. Among various nanoparticles, CdTe quantum dots (QDs) attract great interest due to their excellent optical properties.^4,5^ However, nanoparticles such as CdTe QDs with ultra-small size are easily cleared out through the renal system, resulting in minimal interactions with the diseased area. This problem can be solved by further modification.^6,7^ Loading CdTe QDs into a large-sized biocompatible system is one of the most common methods, in which CdTe QDs still maintain their original structure and optical properties. Silica is classified as “generally recognized as safe” (GRAS) by the FDA and used frequently in cosmetics and as a food-additive. Due to its advantages, such as a stable skeleton structure, regular and continuously adjustable aperture and active surface properties, mesoporous silica nanoparticles (MSN) can effectively load and transport cargoes of different sizes and types.^8–11^ As one type of MSN, hollow mesoporous silica nanoparticles (hMSN) with a large cavity in the structure have also attracted significant attention for their ultra-high cargo holding capacity.^12,13^

Blood vessel growth (angiogenesis) is critical for tissue growth, development and remodeling. However, it is also a fundamental step in the transition of tumors from a benign state to a malignant one. When the tumor reaches a certain size (generally 1–2 mm), angiogenesis can occur to supply enough oxygen and essential nutrients.^14,15^ Tumors induce angiogenesis by secreting various growth factors and proteins. As one of the blood vessel growth factors and a key regulator in the development of tumor, vascular endothelial growth factor (VEGF) and VEGF receptor (VEGFR) pathway in tumor angiogenesis has attracted the interest of researchers in the field of cancer research and therapy.^16–18^ VEGF is mainly concentrated around the tumor vessels, and the response of tumor vessels to VEGF is higher than that of normal vessels, suggesting that VEGF is closely related to tumor angiogenesis. Overexpression of VEGF is associated with the development of multiple tumors and malignant prognosis in colon, breast, prostate and lung cancers. Previous studies have shown that the development of cervical cancer is related to VEGF expression directly.^19–21^ Therefore, the expression specificity of VEGF can result in superior contrast for cancer detection, which makes it an ideal candidate for image-guided drug delivery *via* nanomaterials.

Targeting of angiogenic markers on tumor vasculature has been accepted as a generally applicable strategy for various nanomaterials regardless of the tumor type. Here, a target recognition and drug delivery system of CdTe@hMSN(DOX)–VEGF Abs was established using CdTe QDs as the fluorophore, hMSN as the drug delivery system, and doxorubicin (DOX) as the model drug. The optical and structural properties of the composite nano-system were studied, and targeting recognition was also confirmed. *In vitro* assays (*e.g.*, flow cytometry and confocal fluorescence microscopy) were carried out to validate the target recognition as well as the cytotoxic effect of the released DOX. *Ex vivo* experiments (*e.g.*, distribution and histology) were performed on mice bearing HeLa tumors to demonstrate VEGF specificity of VEGF Abs-conjugated CdTe@hMSN. From these experimental data, we inferred that intrinsic CdTe@hMSN(DOX)–VEGF Abs showed great potential to be used for future targeted cancer therapy.

## Experimental

### Chemicals and materials

Hexadecyl trimethyl ammonium chloride (CTAC) and 3-aminopropyltrimethoxysilane (APS) were purchased from Braunwell Chemical Technology Co., Ltd. Tellurium powder, cadmium chloride, glutathione (GSH), sodium borohydride, tetraethyl orthosilicate (TEOS), triethanolamine (TEA), doxorubicin hydrochloride (DOX·HCl), and sodium hydroxide were purchased from Shanghai Aladdin Biochemical Technology Co., Ltd. Sodium carbonate, sodium chloride, methanol, and anhydrous ethanol were acquired from Sinopharm Group Chemical Reagent Co., Ltd. Fetal bovine serum (FBS) was acquired from Gibco Biochemical Technology Co., Ltd. 3-(4,5-Dimethylthiazol-2-yl)-2,5-diphenyltetrazolium bromide (MTT), dimethyl sulfoxide (DMSO) and 6-diamidino-2-phenylindole (DAPI) were purchased from Nanjing KeyGen Biotechnology Inc. Dulbecco's modified Eagle's medium (DMEM), roswell park memorial institute medium (RPMI)-1640, and trypsin were purchased from Vicmed (Xuzhou, China). SCM-PEG-Mal (*i.e.,* succinimidyl carboxymethyl PEG maleimide, MW: 5 kDa), Traut's reagent, tris(2-carboxyethyl)phosphine (TCEP) reagent were acquired from Thermo Fisher Scientific, Inc. VEGF Abs was acquired from Abcam Biochemical Technology Co., Ltd. Cy3-labeled donkey anti-rat IgG was purchased from Beijing Boao Sen Biotech Co., Ltd. All buffers were prepared using Millipore-grade water. HeLa and L929 cells were provided by Jiangsu Key Laboratory of New Drug Research and Clinical Pharmacy. All reagents were directly used without further purifications following manufacturer's instructions.

### Synthesis of CdTe@hMSN nanoparticles

CdTe QDs were prepared using a previously published method with GSH as the stabilizer, where the molar ratio of Cd : Te : GSH was 2 : 1 : 3.^22,23^ The CdTe QDs with maximum emission wavelength of 550 nm were acquired after refluxing the precursor at 100 °C for 3 h. CdTe QDs were precipitated using isopropanol to obtain the powder. The core/shell CdTe@hMSN were synthesized using a modified Stöber method^24^ and Na_2_CO_3_-etching process.^25,26^ Briefly, the prepared CdTe QDs (5 mg) were first dissolved in 20 mL of 1 : 1 mixture of methanol and water (pH value adjusted with 0.4 mL ammonia), followed by addition of 0.5 mL TEOS and allowed to react for 1 h at room temperature (RT) to form CdTe&SiO_2_. After sequentially washing with ethanol and water, CdTe&SiO_2_ was redispersed in 10 mL of water. Then, 1 g CTAC and 100 μL TEA (10%) were thoroughly mixed in 10 mL water, followed by addition of the prepared CdTe&SiO_2_ solution for reaction at RT for 1.5 h. Without any treatment, 80 μL TEOS was added dropwise into the solution to allow the mixture to react at 80 °C for 1 h to form CdTe&SiO_2_@SiO_2_ NPs. Using Na_2_CO_3_ as the etching reagent, 318 mg of Na_2_CO_3_ was added to etch the inner dSiO_2_ at 50 °C for 30 min. The core/shell CdTe@hMSN powder was obtained after washing with NaCl methanol (1%) extraction to remove CTAC. Finally, APS was hydrolyzed for further functionalization with amino-groups to form CdTe@hMSN–NH_2_. Here, the as-prepared CdTe@hMSN was first dispersed in 10 mL absolute ethanol, followed by addition of 0.5 mL APS and the reaction was maintained at 86–90 °C in a water bath for 24 h. Afterwards, the mixture was washed with absolute ethanol three times to remove any residual APS. CdTe@hMSN–NH_2_ was dissolved in water and amine group concentration was determined using a Kaiser test kit.

### CdTe@hMSN functionalization

VEGF-Abs was mixed with Traut's reagent at a molar ratio of 1 : 30 at pH 8.0 for the incorporation of thiols onto the antibody molecules. After 2 h of incubation at RT, the resulting VEGF-Abs-SH was purified by PD-10 column using phosphate-buffered saline solution (PBS) as the mobile phase. Based on the titration results from Ellman's reagent, we calculated that there were 5 thiol groups per VEGF-Abs on an average under this reaction condition.

CdTe@hMSN–NH_2_ was reacted with SCM-PEG_5k_-MAL at pH 8.5 at a molar ratio of 1 : 25 for 2 h. After removing unreacted SCM-PEG_5k_-MAL by purifying with 50k Amicon filter, CdTe@hMSN-PEG-MAL was obtained. Subsequently, CdTe@hMSN-PEG-MAL was mixed with VEGF-Abs-SH at a molar ratio of 1 : 6 at pH 8.0 in the presence of TCEP to protect thiols from oxidation. After reacting for 12 h at 4 °C, the final product CdTe@hMSN–VEGF Abs was collected by PD-10 purification.

### Material characterization

The morphology of CdTe@hMSN conjugates was evaluated by G2T12 transmission electron microscopy (TEM) (FEI, USA). Meanwhile, their hydrodynamic size and size distribution as well as ζ-potentials were determined by a 380 ZLS dynamic light scattering (DLS) instrument (Nicomp, USA). The loading amount of CdTe QDs in hMSN and probable cadmium ion release were measured by inductively coupled plasma atomic emission spectroscopy (ICP-AES) (PE, USA). Ultraviolet-visible (UV-Vis) and emission spectra assays were carried out on Hitachi U-3010 and F-4600 fluorescence spectrometers (Hitachi, Japan), respectively.

### Cell cytotoxicity assay of CdTe@hMSN conjugates

Cell cytotoxicity analysis was performed using MTT assay. HeLa and L929 cells were seeded in a 96-well plate at a density of 6 × 10^3^ cells per well. After separate incubations with CdTe@hMSN–NH_2_, CdTe@hMSN-PEG, and CdTe@hMSN–VEGF Abs for 24 h, the relative viabilities of cell samples were determined by a cell titer following the vendor's protocols. The percentages of viable cells relative to the untreated control were plotted against CdTe@hMSN conjugate concentrations.

### Drug loading and releasing measurement

DOX was used as a model drug to test the drug loading capacity of CdTe@hMSN. DOX·HCl aqueous solution was adjusted to pH 8.0 with TEA, followed by mixing with CdTe@hMSN conjugate (CdTe@hMSN or CdTe@hMSN–VEGF Abs) at a mass ratio of 1 : 1 based on the amount of CdTe@hMSN. The mixture was stirred for 24 h at RT, and excess DOX was removed by filtration through a 2 kDa filter with repeated rinsing of PBS. The drug release was evaluated at 37 °C in simulated physiological condition at the pH values of 5.0, 6.5, and 7.4. CdTe@hMSN(DOX)–VEGF Abs or CdTe@hMSN(DOX) was placed in a dialysis bag with a molecular weight cut-off of 2 kDa. The dialysis bag was immersed in the release medium and kept in a shaker (100 rpm) under RT. Samples of 0.2 mL volume were periodically removed and the same volume of fresh medium was added. The loading and releasing rates of DOX in CdTe@hMSN(DOX)–VEGF Abs and CdTe@hMSN(DOX) were calculated by determining unbound DOX in the washing/releasing solutions by UV-visible spectrometry. The DOX release studies were performed in triplicate for each sample.

### Flow cytometry and fluorescence microscopy

HeLa and L929 cells were harvested and suspended in PBS buffer (supplemented with 2% bovine serum albumin) at a concentration of 2 × 10^6^ cells per ml, incubated with CdTe@hMSN and CdTe@hMSN–VEGF Abs (2 μg mL^−1^ based on CdTe@hMSN) for 1 h at 37 °C, and washed three times with PBS. Afterwards, the cells were resuspended in PBS and analyzed using a MACSQuant Analyzer (Miltenyi Biotec GmbH, Germany). Cellular fluorescence was computed *via* FlowJo (X.0.7, Tree Star, Ashland, OR). The cells were also examined under an Olympus FV10i (Olympus, Japan) confocal microscope with 200× magnification to validate the flow cytometry results.

### *In vivo* tumor accumulation

All animal procedures were performed in accordance with the National Academy of Sciences Guide for the Care and Use of Laboratory Animals of USA ^27^ and approved by the Animal Ethics Committee of Xuzhou Medical University. Tumors were established by subcutaneous injection of 2 × 10^6^ of HeLa cells suspended in 100 μL of PBS into the female nude mice. The tumor sizes were monitored every other day and the mice were subjected to imaging studies when the tumor diameter reached 5–8 mm. The mice were sacrificed at 2 h p.i., and the tumor and muscle were removed for *ex vivo* fluorescence imaging in the Berthold LB983 NightOWL II system (485/600 nm for DOX). To confirm the *in vivo* tumor accumulation of CdTe@hMSN conjugates, frozen tumors were cut into slices of 6 μm thickness. After being fixed with cold acetone for 10 min, tumors were rinsed with cold PBS and blocked with 2% BSA for 30 min. Subsequently, the tissue slides were stained for endothelial marker CD31 with a rat anti-mouse CD31 antibody (2 μg mL^−1^) for 1 h, followed by Cy3-labeled donkey anti-rat IgG (3 μg mL^−1^) for 2 h. The locations of CdTe@hMSN conjugates were visualized using the fluorescence of CdTe QDs (which emitted at 550 nm under 350 nm excitation). All fluorescence images were taken with an IX73 digital microscope (Olympus, Japan) with 200× magnification.

## Results and discussion

### Material characterization

**Fig. 1:**
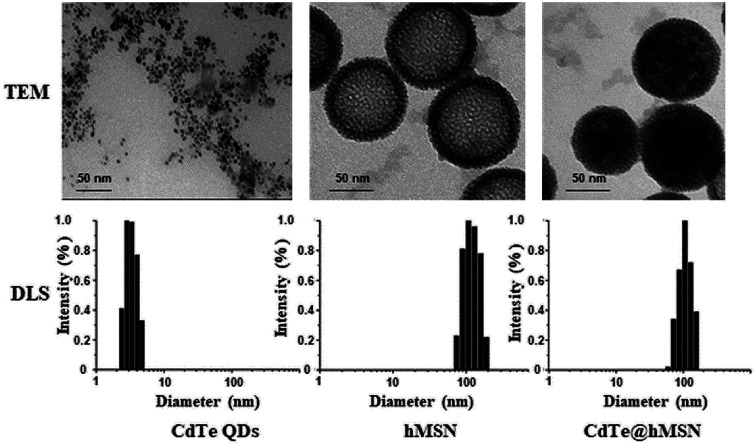
The morphology of CdTe QDs, hMSN, and CdTe@hMSN.

CdTe@hMSN were found to be core/shell nanocrystals with CdTe QDs as the core and hMSN as the shell. Based on TEM measurements in Fig. 1, the individual sizes of CdTe QDs, hMSN, and CdTe@hMSN were about 3.0 ± 1 nm, 90 ± 10 nm, and 90 ± 10 nm, respectively. The sizes obtained from DLS curve were larger than that obtained from TEM images, which resulted from the hydration of NPs in aqueous solution. By measuring the total amount of Cd (from CdTe QDs) using ICP-AES testing, the loading efficiency of 0.15 mg CdTe QDs/mg hMSN was calculated.

**Fig. 2:**
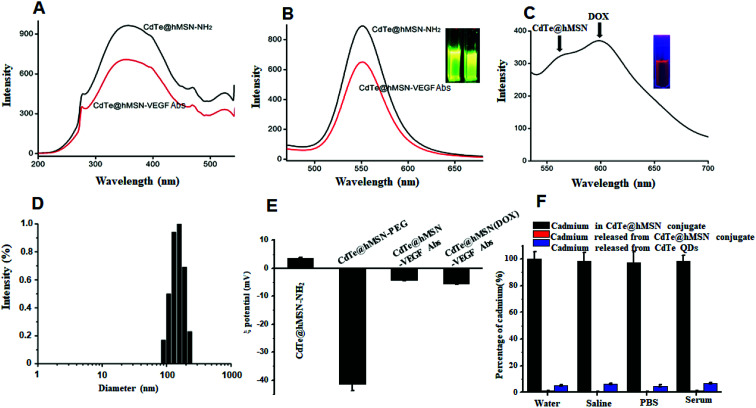
Characteristics of CdTe@hMSN conjugates. (A) Excitation spectra of CdTe@hMSN conjugates; (B) emission spectra of CdTe@hMSN conjugates; (C) emission spectra of CdTe@hMSN(DOX)–VEGF Abs; (D) DLS curve of CdTe@hMSN–VEGF Abs; (E) ζ-potential of CdTe@hMSN conjugates; (F) ICP-AES testing of cadmium release.

CdTe@hMSN was modified with APS hydrolysate to form amine groups on the surface. The amine group concentration determined by the ninhydrin coloration method was about 200 nmol mL^−1^. After reacting with SCM-PEG_5k_-MAL, it could be linked with an antibody by covalent binding. As shown in Fig. 2A and B, the optical properties such as excitation and emission did not vary after conjugation with the VEGF antibody. Compared with the result for rhodamine 6G (95%), the fluorescence yield of CdTe@hMSN was 12.3%. As shown in Fig. 2C, two fluorescence emission peaks of CdTe@hMSN and DOX confirmed the successful drug loading in CdTe@hMSN(DOX)–VEGF Abs. The DLS results in Fig. 2D also indicated that the size of CdTe@hMSN conjugates increased slightly after surface modification with SCM-PEG_5k_-MAL and VEGF Abs from *ca.* 100 nm (CdTe@hMSN) to *ca.* 110 nm (CdTe@hMSN–VEGF Abs). The successful surface engineering was further validated by ζ-potential measurements, as shown in Fig. 2E, in which a significant change in surface charge was observed after SCM-PEG_5k_-Mal coating (ζ-potential: from 3.49 ± 0.31 mV to −41.40 ± 2.3 mV). After conjugating with VEGF Abs, the surface charge of CdTe@hMSN–VEGF Abs was −4.25 ± 0.21 mV, which was similar to that of CdTe@hMSN(DOX)–VEGF Abs. The probable cadmium ion release rate from CdTe@hMSN was measured by detecting the cadmium concentration using ICP-AES. After incubating CdTe@hMSN in different media for 7 days, the percentages of released cadmium were all lower than 1.5% and were significantly lower than those for naked CdTe QDs. The results indicated that hMSN played an important role in enhancing the stability of CdTe QDs.

### DOX loading and release

**Fig. 3:**
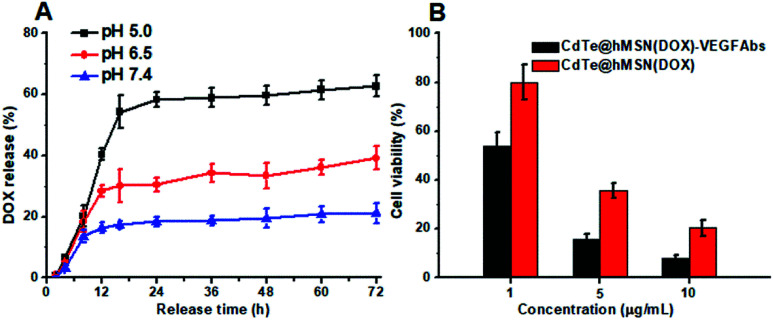
DOX release curves in different media and cell viability test for released DOX. (A) DOX release curves in different media; (B) HeLa cell viability in the presence of CdTe@hMSN conjugates at pH 6.5.

Based on the hollow structure, hMSN is definitely suitable for loading of various cargos. The amount of DOX loaded into the CdTe@hMSN conjugates was calculated to be 0.743 mg mg^−1^ CdTe@hMSN conjugates based on the absorbance measurement at 490 nm for DOX. Under simulated physiological condition at the pH values of 5.0, 6.5 and 7.4 and at 37 °C, the DOX release profile from CdTe@hMSN(DOX) was measured. The results shown in Fig. 3A indicated that the medium pH had an impact on the release rate of DOX from the CdTe@hMSN conjugates. At pH 7.4, 21.2% of DOX was released after 72 h, which suggested that loaded DOX within CdTe@hMSN was relatively stable under physiological condition. In contrast, when medium pH was decreased to 5.0 (mimics endocytic compartments, where the pH ranges from 4.5 to 6.5), the amount of released DOX increased to approximately 62.74% (0.47 mg DOX/mg CdTe@hMSN). The results confirmed that DOX can be loaded into CdTe@hMSN-PEG with high efficiency and the release behavior is pH-dependent. Meanwhile, the effects of CdTe@hMSN(DOX) and CdTe@hMSN(DOX)–VEGF Abs on HeLa cell viability were also tested. Compared with the CdTe@hMSN(DOX) group, CdTe@hMSN(DOX)–VEGF Abs exhibited cytotoxicity (in Fig. 3B). With the addition of target VEGF Abs, the tumor cell death significantly increased, which further verified VEGF Abs specificity and enhanced DOX release.

### *In vitro* tumor cell targeting

**Fig. 4:**
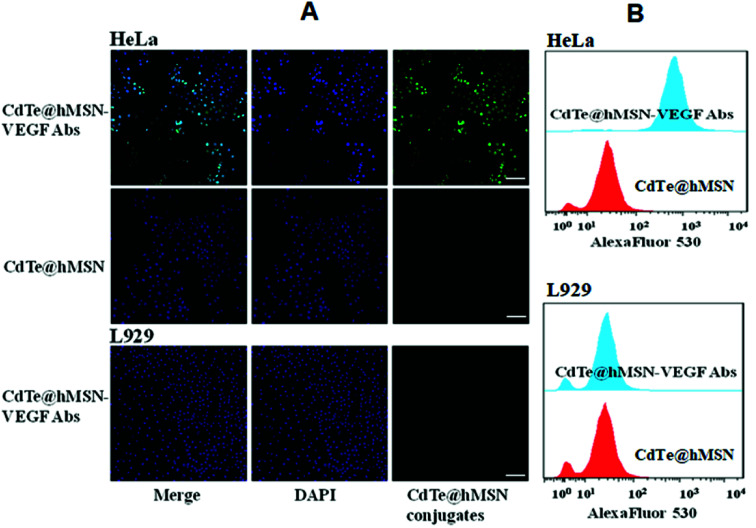
*In vitro* assay of CdTe@hMSN conjugates. (A) confocal fluorescence microscopy evaluation of CdTe@hMSN conjugates; (B) flow cytometry analysis of CdTe@hMSN conjugates.

Two cell lines were used for *in vitro* evaluation of CdTe@hMSN conjugates: VEGF-positive HeLa cells and VEGF-negative L929 cells. Flow cytometry analysis and microscopy studies of CdTe@hMSN and CdTe@hMSN–VEGF Abs were conducted with CdTe QDs as the fluorophore (green color). DAPI staining (blue color) was used to identify the location of nucleus. As shown in the FASC data from Fig. 4B, incubation with 5 μg mL^−1^ CdTe@hMSN–VEGF Abs greatly enhanced the cellular fluorescence intensity compared with that from the CdTe@hMSN group at the same concentration (∼20 fold higher than that of the CdTe@hMSN group). On the contrary, CdTe@hMSN and CdTe@hMSN–VEGF Abs exhibited very minimal specific binding with L929 cells. The VEGF Abs specificity was further validated by confocal fluorescence microscopy evaluation (Fig. 4A). In VEGF-positive HeLa cells, the fluorescence intensity from the CdTe@hMSN–VEGF Abs group was substantially stronger than that from the CdTe@hMSN group. The distribution pattern of DAPI overlapped well with that from CdTe, partly indicating that the nano-system entered the nuclei after cellular internalization. CdTe@hMSN–VEGF Abs did not show a clear cellular fluorescence signal in VEGF-negative L929 cells.

### Enhanced DOX delivery to tumors

**Fig. 5:**
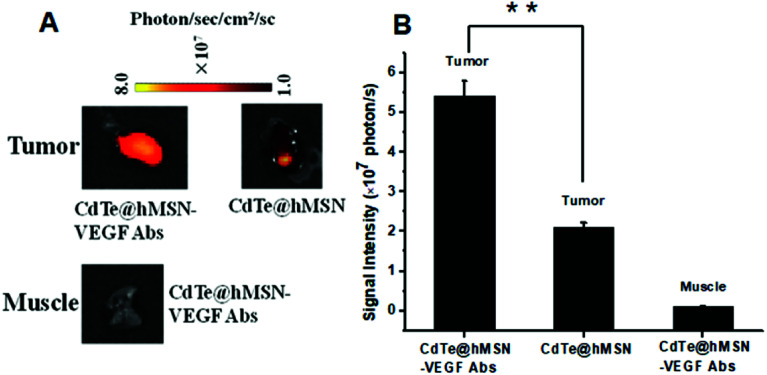
DOX fluorescence imaging and quantification in tumors and muscles. (A) DOX fluorescence imaging in tumors and muscles; (B) DOX quantification in tumors and muscles.

The feasibility of enhanced DOX delivery of CdTe@hMSN–VEGF Abs at tumor sites was studied by *ex vivo* fluorescence since DOX loaded in CdTe@hMSN conjugates exhibited strong fluorescence emission. After intravenous injection of CdTe@hMSN(DOX)–VEGF Abs or CdTe@hMSN(DOX) for 2 h, tumors and muscles were collected and imaged on a NightOWL II system (ex/em = 485/600 nm, where CdTe QDs have no effect on the fluorescence). As shown in Fig. 5A, a strong optical signal from DOX could be observed for CdTe@hMSN(DOX)–VEGF Abs group. Quantitative data from ROI analysis of the tumors and muscles (Fig. 5B) showed significantly higher fluorescent signal of DOX for CdTe@hMSN(DOX)–VEGF Abs, which was about 2.5-fold that for CdTe@hMSN(DOX), clearly demonstrating the feasibility of delivering higher amounts of anticancer drugs to tumor sites *in vivo* using the VEGF antibody-conjugated CdTe@hMSN system.

### Histology

**Fig. 6:**
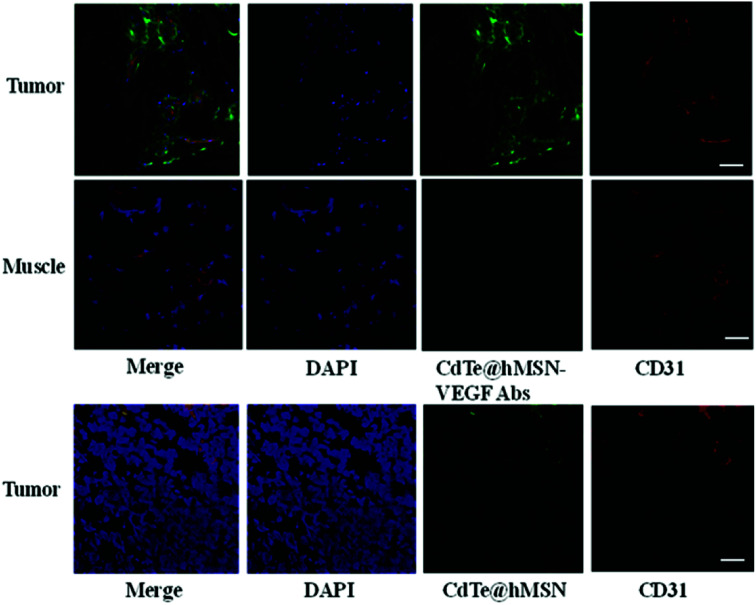
Immunofluorescence staining of tumor nodules and muscles.

To further confirm high accumulation of CdTe@hMSN conjugates in tumors, histological studies were carried out. Mice injected with CdTe@hMSN–VEGF Abs and CdTe@hMSN were euthanized at 2 h p.i. and then, HeLa tumor nodules and muscles were frozen and cryo-sectioned for immunofluorescence staining. Tumors treated with CdTe@hMSN–VEGF Abs and CdTe@hMSN were frozen for histological analysis. With negligible CdTe@hMSN conjugate uptake, the muscle tissue was also examined as a normal control. The green fluorescence in Fig. 6 is ascribed to the presence of CdTe@hMSN conjugates as CdTe exhibits emission at 550 nm. Red fluorescence was from CD31, a vasculature marker. From the green fluorescence intensity and distribution, a substantial amount of CdTe@hMSN–VEGF Abs was accumulated in HeLa tumors, which was significantly higher than that for the CdTe@hMSN group. CdTe@hMSN–VEGF Abs was primarily located on the tumor vasculature, which indicated that tumor vasculature-targeting is truly responsible for the enhanced tumor uptake of CdTe@hMSN conjugates. However, the partial overlap of green and red fluorescence (which delineates CD31) indicated some extravasation of CdTe@hMSN conjugates from vasculature at that time. Overlap of green and blue fluorescence verified that CdTe@hMSN conjugates could enter the nuclei, which was consistent with the results from cell staining. No observable green fluorescence was detected in muscles.

## Conclusion

In conclusion, a core/shell CdTe@hMSN–VEGF Abs system was designed and synthesized for enhanced drug delivery and accumulation in tumors. The optical properties of CdTe QDs from CdTe@hMSN conjugates remain stable due to the protection of hMSN, which could be used for immunostaining directly without further modifying with fluorescence dye. Relatively high amount of DOX could be loaded in CdTe@hMSN conjugates, and pH-dependent DOX release behavior was detected. Relying on the VEGF target, the accumulation of CdTe@hMSN conjugates and enhanced DOX delivery to HeLa tumors *in vivo* were demonstrated in tumor-bearing mice. A problem that cannot be ignored in this study is that CdTe QDs are not optimal materials due to their potential toxicity and limited fluorescence emission wavelength. This has encouraged us to develop biomedical agents with higher biocompatibility and longer emission wavelength in the future study.

## Conflicts of interest

There are no conflicts to declare.

## Supplementary Material
